# *Bifidobacterium longum*, *Lactobacillus plantarum* and *Pediococcus acidilactici* Reversed ETEC-Inducing Intestinal Inflammation in Mice

**DOI:** 10.3390/microorganisms10122350

**Published:** 2022-11-28

**Authors:** Wentao Li, Lixia Kai, Zipeng Jiang, Huan He, Mingzhi Yang, Weifa Su, Yizhen Wang, Mingliang Jin, Zeqing Lu

**Affiliations:** 1 National Engineering Research Centre for Green Feed and Healthy Farming, Zhejiang University, 866 Yuhang Tang Road, Hangzhou 310058, China; 2 Key Laboratory of Molecular Nutrition, Ministry of Education, Zhejiang University, 866 Yuhang Tang Road, Hangzhou 310058, China; 3 Key Laboratory of Animal Nutrition and Feed, Ministry of Agriculture and Rural Affairs, Zhejiang University, 866 Yuhang Tang Road, Hangzhou 310058, China; 4 Key Laboratory of Animal Nutrition and Feed Nutrition of Zhejiang Province, Zhejiang University, 866 Yuhang Tang Road, Hangzhou 310058, China; 5 Institute of Feed Science, College of Animal Science, Zhejiang University, 866 Yuhang Tang Road, Hangzhou 310058, China

**Keywords:** microecological preparation, ETEC, *Bifidobacterium longum*, *Lactobacillus plantarum*, *Pediococcus acidilactici*, inflammation, intestinal microbiota

## Abstract

Microecological preparation could relieve Enterotoxigenic Escherichia coli (ETEC) K88-induced diarrhea in piglets, but which bacteria play a key role and the mitigation mechanism have not been fully clarified. In this study, 36 male mice were randomly divided into six groups (CON, K88, BK (*Bifidobacterium longum* + K88), LK (*Lactobacillus plantarum* + K88), PK (*Pediococcus acidilactici* + K88), and MK (mixed strains + K88)) to explore the prevention mechanisms. Three probiotic strains and their mixtures (TPSM) significantly relieved the weight loss and restored the ratio of villus height to crypt depth in the jejunum. Except for Bifidobacterium longum, other strains significantly decreased interleukin (IL)-1β, IL-6 and tumor necrosis factor-α (TNF-α) in mice serum. The TPSM treatment significantly downregulated the mRNA expression of the inflammatory cytokines and the Toll-like receptor and downstream gene (*TLR4*, *MyD88*, *NF-κB*) in jejunum induced by ETEC. Furthermore, the TPSM could restore dysbiosis of the intestinal microbiota caused by ETEC. The intestinal microbiota analysis demonstrated that *Bifidobacterium longum* enriched the *Bifidobacterium* genus (*p* < 0.05), *Lactobacillus plantarum* enriched the *Lactobacillus* genus (*p* < 0.05), *Pediococcus acidilactici* enriched the *Coriobacteriaceae_UCG-002* and *Christensenellaceae_R-7_group* genus (*p* < 0.05), mixed bacteria enriched the *Akkermansia* genus (*p* < 0.05), but ETEC enriched the *Desulfovibrio* genus (*p* < 0.05). Meanwhile, the starch and sucrose metabolism, galactose and fructose metabolism, mannose metabolism and ABC transporters were increased with probiotics pre-treatment (*p* < 0.05). To sum up, the microecological preparation alleviated ETEC-induced diarrhea by regulating the immune response, rebalancing intestinal microbiota and improving carbohydrate metabolism.

## 1. Introduction

*Enterotoxigenic Escherichia coli* (ETEC) is a potential zoonotic pathogen that causes extensive diarrhea in children and tourists [1,2]. Approximately 3–5 million children lose their life due to diarrhea annually worldwide under the age of five, especially in developing countries [3]. Similarly, diarrhea resulting from ETEC is also a great threat to newborn animals, particularly in piglets, whose mortality is up to 50% [4]. ETEC produces heat-labile and heat-stable enterotoxins, which destroy the intestinal epithelial barrier and disturb the intestinal water–salt metabolism [5]. With high morbidity and mortality, ETEC K88 is the main serotype threatening the health of newborn and weaned piglets [6]. It has caused great economic losses all over the world [7]. K88 fimbriae can help bacteria target the corresponding receptors of the host’s small intestinal mucosal epithelial cells, then colonize and produce toxins [8], thereby damaging the host’s intestinal mucosa, disturbing the host’s intestinal microbiota [9], hindering abnormal absorption and secretion function, and finally lead to diarrhea [10].

Antibiotic overuse came with large side effects, such as livestock antibiotics residues, human antibiotic-associated diarrhea and bacterial drug resistance. Numerous studies have indicated that probiotics [11], prebiotics [12], enzyme preparations [13], acidifiers [14], plant extracts [15], and antimicrobial peptides [16] can effectively reduce the use of antibiotics. Probiotics could not only secrete organic acids and bacteriocins in the intestine [17,18] but also compete with pathogens for colonization sites [19] and nutrients and growth factors [20], which further inhibit the growth and reproduction of pathogens and maintain the normal composition of intestinal microbiota. Bifidobacteria and lactic acid bacteria are important Autochthonous Microbiota in human and animal intestines [21]. In long-term co-evolution, the host does not produce an immune response to them [22], and they are considerable probiotics that play a vital role in resisting pathogens in young animal health [23]. For example, *Lactobacillus rhamnosus* LB1 improves piglet intestinal health and alleviates ETEC-induced diarrhea and mortality in piglets by modulating intestinal function-related proteins and genes [24]. Moreover, both *Bifidobacterium bifidum* FSDJN7O5 and *Bifidobacterium breve* FHNFQ23M3 alleviate diarrhea by downregulating TNF-α, restoring the structure of jejunal villi, increasing the SCFA content, and altering intestinal microbiota disorders caused by ETEC [25]. Furthermore, *Pediococcus acidilactici* P25 inhibits the proliferation of ETEC K88 by altering the intestinal pH through the secretion of organic acids [26]. Our group found that newborn piglets gavaged with the microecological preparation (containing *Bifidobacterium longum*, *Lactobacillus plantarum* and *Pediococcus acidilactici*) had lower diarrhea rates compared to those without the microecological preparation (unpublished data). Nevertheless, the mitigative effect and mechanism of the microecological preparations on diarrhea still need to be further studied. In the present study, a mouse model was constructed to investigate which bacteria in the microecological preparation played a key role in preventing diarrhea by ETEC K88. The aim of this study is to compare the effectiveness and mechanism of three probiotic strains and their mixture (TPSM) in preventing ETEC K88 in mice, thus providing theoretical support for the microecological preparation to prevent ETEC-induced piglets’ diarrhea.

## 2. Materials and Methods

### 2.1. Bacterial Strains Preparation

The *Bifidobacterium longum subsp. Infantis* LR655210.1, *Lactobacillus plantarum* ON329060.1, and *Pediococcus acidilactici* MW882989.1 were purchased from Evolve Biosystems Inc. (Davis, CA, USA). *Bifidobacterium longum* subsp. *Infantis* was cultured in a TPY medium and added to skimmed milk powder (Inner Mongolia Yili Industrial Group Limited by Share, Huhehot, China). *Lactobacillus plantarum* and *Pediococcus acidilactici* were cultured in an MRS medium. *Bifidobacterium longum* subsp. *Infantis* was strictly static-anaerobic cultured in an anaerobic incubator at 37 °C for 36 h, while the other two bacteria were for 12 h. Then, they were used for experiments in the logarithmic growth stage (absorbance under 540 nm = 0.8), which had strong growth and reproduction abilities. The standard Escherichia strain O60:K88 producing heat-labile toxins was isolated from diarrheal piglets. After overnight incubation at 37 °C in a shaking incubator, bacteria were diluted in a fresh LB broth at a ratio of 1:100 and shaken for 3 h until the mid-logarithmic phase (absorbance under 540 nm = 0.5).

All strains were centrifuged at 3000× *g* for 10 min at 4 °C and resuspended in phosphate-buffered saline (PBS) following incubation. The final concentration of all strains was 3.0 × 10^8^ bacterial colony-forming units (CFU) per milliliter.

### 2.2. Animal Experimental Design

**Figure 1:**
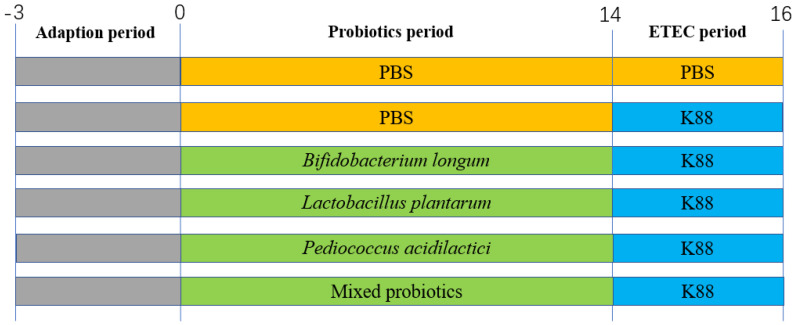
Animal design.

The animal experiment was approved by the Zhejiang University Animal Research Ethics Committee (No. ZJU20220164) and conducted in accordance with the National Institutes of Health guidelines. Healthy male C57BL/6 mice were obtained from the Shanghai Laboratory Animal Co., Ltd. (SLAC, Shanghai, China) at the age of 4 weeks. A total of 36 male mice were randomly divided into six groups (CON, K88, BK (*B. longum* + K88), LK (*L. plantarum* + K88), PK (*P. acidilactici* + K88), and MK (*Mixed strains* + K88)) and fed in a room at 24 °C with 12 h/d lighting times and a relative humidity of 45–55%. After a three-day adaptation period, each group was tested, as shown in Figure 1: the CON group was gavaged with PBS (200 μL) from days 0 to 16 once a day; the K88 group was gavaged with PBS (200 μL) from days 0 to 14, then, they intragastric accepted ETEC K88 (200 μL) once every 12 h from days 14 to 16 for three times in total; similar to the K88 group, the remaining four groups were gavaged with probiotics or their mixtures from days 0 to 14, and oral administration of ETEC K88, as previously described. The gavage concentration of all probiotics and ETEC K88 were 3.0 × 10^8^ CFU/mL. The body weight and food intake were assessed every three days.

### 2.3. Sample Collection and Treatment

Mice were sacrificed after blood collection from the eyeballs at 9:00 a.m., 12 h after the last gavage. The blood was centrifuged at 3000× *g* for 10 min at 4 °C to prepare the serum. Secondly, we weighed the spleen and measured the colon with a Vernier caliper. Then, the segment (2 cm) of jejunum was dissected after washing the intestinal contents with cold, sterile PBS, which was used for hematoxylin-eosin (H&E) staining and gene expression determination. The contents of the colon were collected for 16 s microbial diversity analysis.

### 2.4. Detection of Inflammatory Cytokines and Immunoglobulin in Mice Serum

The levels of IL-1β, IL-6, TNF-α, IgA, IgM and IgG in serum were measured using ELISA kits (Jiangsu Enzyme-Labeled BioTECH, Yancheng, China) and then by operating other steps following the protocol according to the manufacturer’s instruction.

### 2.5. Intestinal Morphology Analysis

The jejunum samples were collected and fixed by 4% formaldehyde, embedded in paraffin, and stained with H&E. The vitalizations were captured under a light microscope (Leica DM3000, Wetzlar, Germany).

### 2.6. RNA Extraction and Quantitative Real-Time PCR

Following the manufacturer’s guidelines, total RNA was extracted from the jejunum tissues of mice by using TRIzol reagent (Invitrogen Life Technologies, Waltham, MA, USA). The concentration and purity of the RNA were measured using NanoDrop2000 (Thermo Scientific, Wilmington, NC, USA). Then, 2 μg of RNA was used to reverse transcribe into the cDNA with the random primers in Table 1. The ABI StepOnePlus real-time PCR system (Applied Biosystems, CA, USA) was used to analyze the cDNA using the Fast Strat Universal SYBR Green master mix (Roche, Mannheim, Germany). We measured the mRNA expression of the target gene using the comparative CT method (2^−∆∆Ct^ method), and the results were normalized by using β-actin as the housekeeping gene.

### 2.7. Microbial Analysis of Colon Contents

Total genomic DNA was extracted from the colon content samples using the QIAamp DNA Stool Mini Kit (QIAGENLtd., Hilden, Germany). The primer sequences 338F: 5′-ACTCCTACGGGAGGCAGCAG-3′ and 806R: 5′-GGACTACHVGGGTWTCTAAT-3′ were used to amplify the V3-V4 region of the bacterial 16S rRNA gene. Then, a PCR reaction was performed as follows: 3 min of denaturation at 95 °C, then 27 cycles of 30 s each at 95 °C, 15 s to anneal at 55 °C, 15 s to elongate at 72 °C, and 10 min to the final extension at 72 °C. The PCR products subsequently were extracted, purified, and quantified using the QIAquick gel extraction kit (QIAGEN, Hilden, Germany) and Quant-iT PicoGreen dsDNA assay kit (Life Technologies, Carlsbad, CA, USA). For the next step, the 16S rRNA libraries were sequenced using an Illumina HiSeq2500 (Novogene, China) according to previous studies [27,28,29]. As a final step, the data were processed using the QIIME package (V1.7.0, http://qiime.org/scripts/split_libraries_fastq.html) following MiSeq genome sequencing.

### 2.8. Statistical Analysis

The SPSS 26.0 software was applied for the data analysis. A one-way ANOVA analysis was used to test the differences among groups. Bar plots were generated in GraphPad Prism 8.02. Data were expressed as the mean ± SD. A *p* < 0.05 was considered statistically significant.

## 3. Results

### 3.1. Effect of Probiotics Administration on Growth Performance of Mice

**Figure 2:**
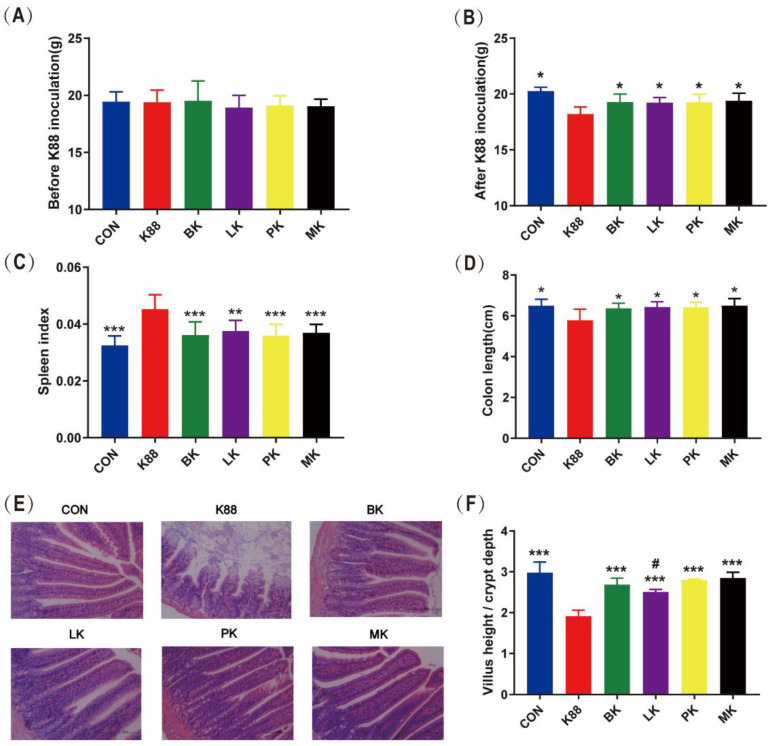
Effect of probiotics on the symptoms of ETEC K88-induced mice. (**A**) Before K88 inoculation (day 14), (**B**) after K88 inoculation (day 16), (**C**) spleen index, (**D**) colon length, (**E**) jejunal morphology (scale bars: 100 μm), (**F**) villus height/crypt depth. * represents significant differences vs. K88 group (*: *p* < 0.05, **: *p* < 0.01, ***: *p* < 0.001) ^#^ represents significant differences vs. CON group (^#^: *p* < 0.05,). All data are presented as the mean ± SD.

The body weight was recorded every three days. Moreover, the body weight was recorded before gavage ETEC, and the last weight record was close to the mice sacrificed. A significant reduction in body weight was observed in the K88 group after a challenge with ETEC K88 from day 14 to day 16; however, the trend in weight loss was alleviated in the BK, LK, PK and MK groups. In particular, the body weight of CON, K88, BK, LK, PK and MK on day 16 was 20.66 ± 0.27 g, 18.22 ± 0.25 g, 19.29 ± 0.27 g, 19.23 ± 0.17 g, 19.27 ± 0.23 g and 19.39 ± 0.23 g, respectively. Body weights decreased significantly (*p* < 0.05) for the K88 group compared to the CON group, but not for BK, LK PK and MK (Figure 2A,B). The spleen index (spleen weight/body weight) is shown in Figure 2C. The K88 group was approximately 1.5 times over that of the CON group and other groups (*p* < 0.05). Although there was a significant decrease in colon length in the K88 group (*p* < 0.05) compared with the CON group, there was no significant difference between the BK, LK, PK or MK groups (Figure 2D).

### 3.2. Probiotics Recovered the Jejunum Tissue Damage

The jejunum morphology and ratio of the villus height to crypt depth (villus height/crypt depth) were measured in order to evaluate the improvement of BK, LK, PK and MK on the jejunal injury caused by ETEC K88. Compared with the CON group, the jejunum of the K88 group was seriously disordered in the villi and crypt structures, while there was no obvious change in the jejunum morphology of the BK, LK, PK or MK groups (Figure 2E). The result displayed that ETEC K88 significantly reduced the villus height/crypt depth ratio of the jejunum, shortening them by 35.91% compared with the CON group (*p* < 0.001); however, the BK, LK, PK and MK treatments significantly increased the ratio (Figure 2F). Meanwhile, compared with the CON group, the LK group significantly decreased the ratio (*p* < 0.05).

### 3.3. Effects of Probiotics on Inflammatory Cytokines and Immunoglobulin in Mice Serum

**Figure 3:**
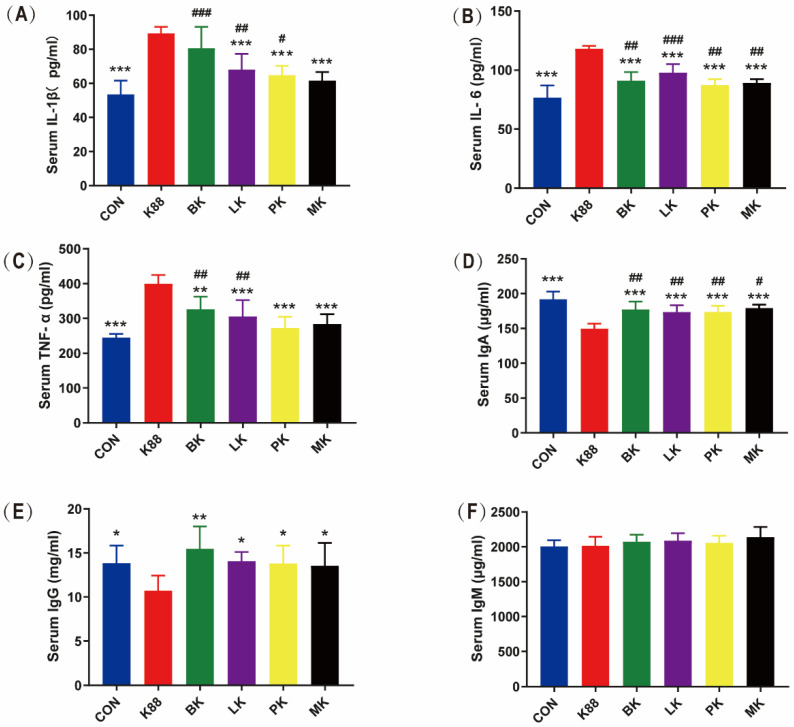
Effects of probiotics on inflammatory cytokines and immunoglobulin in serum of mice. (**A**) IL-1β, (**B**) IL-6, (**C**) TNF-α, (**D**) IgA, (**E**) IgG, and (**F**) IgM. * represents significant differences vs. K88 group (*: *p* < 0.05, **: *p* < 0.01, ***: *p* < 0.001) ^#^ represents significant differences vs. CON group (^#^: *p* < 0.05, ^##^: *p* < 0.01, ^###^: *p* < 0.001). All data are presented as the mean ± SD.

As shown in Figure 3A–C, the K88 group significantly increased IL-1β, IL-6 and TNF-α by 1.67, 1.54 and 1.63 times than that of the CON group (*p* < 0.001). Compared with the K88 group, LK, PK and MK significantly decreased IL-1β, IL-6 and TNF-α (*p* < 0.001), respectively, and BK significantly reversed these enhancements in IL-6 and TNF-α (*p* < 0.001) but did not in IL-1β. Compared with the CON group, the ETEC K88 infection increased the concentration of IL-1β in the BK, LK and PK groups; IL-6 in the BK, LK, PK and MK groups; TNF-α in the BK and LK groups. Meanwhile, the immunoglobulin (Ig) in serum was determined, as shown in Figure 3D–F, and compared with the CON group, the IgA and IgG in the K88 group significantly decreased by 1.28 and 1.29 times. This was the same as the K88 group, where the IgA in the BK, LK, PK and MK groups was also significantly decreased. Compared with the K88 group, the BK, LK, PK and MK groups could significantly increase IgA and IgG. Interestingly, there was no significant difference among the six groups of IgM.

### 3.4. Effects of Probiotics on Inflammatory Cytokines and Toll-like Receptor mRNA Expression in Jejunum of Mice

**Figure 4:**
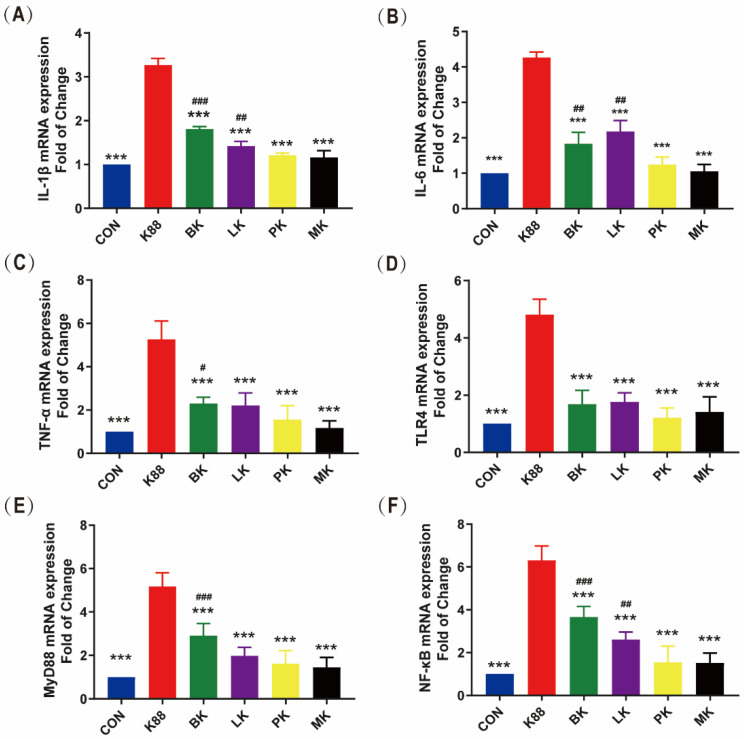
Effects of probiotics on inflammatory cytokines and Toll-like receptor mRNA expression in jejunum of mice. (**A**) IL-1β, (**B**) IL-6, (**C**) TNF-α, (**D**) TLR4, (**E**) MyD88, (**F**) NF-κB. * represents significant differences vs. K88 group (***: *p* < 0.001) ^#^ represents significant differences vs. CON group (^#^: *p* < 0.05, ^##^: *p* < 0.01, ^###^: *p* < 0.001). All data are presented as the mean ± SD.

TLRs belong to an important type of PPRs, which are ubiquitous on the surface or inside a variety of cells in the body and play a key role in recognizing microorganisms by the innate immune system [30]. TLRs can induce the expression of pro-inflammatory cytokines through the MyD88-dependent pathway to activate NF-κB [31]. It can be seen from Figure 4 that the mice treated with ETEC K88 showed acutely elevated expression levels involved in the jejunum mucosal inflammatory response, including IL-1β, IL-6, TNF-α, TLR4, MyD88 and NF-κB (*p* < 0.001). The administration of probiotics dramatically reversed these increments (*p* < 0.001). Compared with the CON group, the expression levels of IL-1β, IL-6, TNF-α, MyD88 and NF-κB in the BK group were significantly increased, and IL-1β, IL-6 and NF-κB in the LK group increased.

### 3.5. Probiotics Modulated Intestinal Microbiota Composition Wrecked by K88

**Figure 5:**
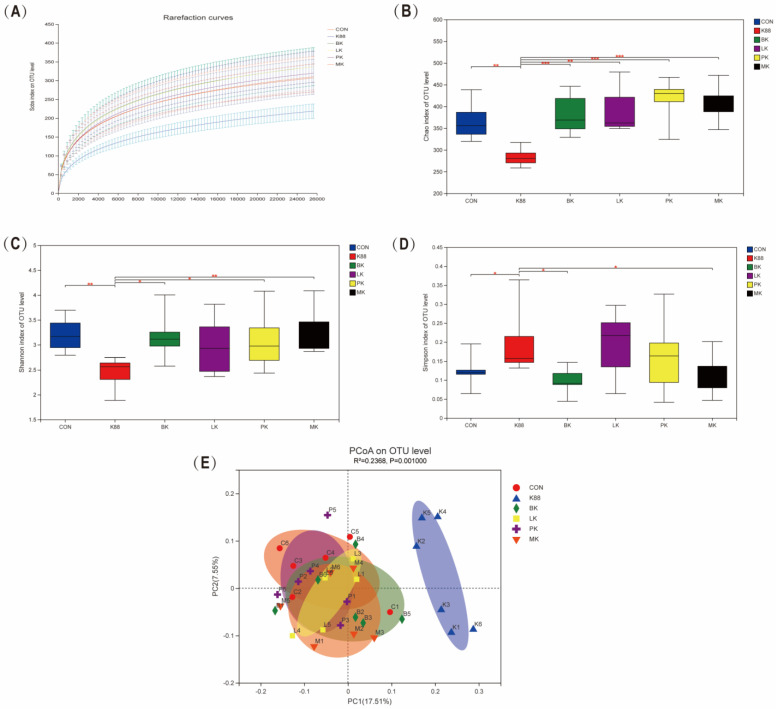
Effect of probiotics on the diversity of intestinal microbiota. (**A**) Rarefaction curves, (**B**) Chao index, (**C**) Shannon index, (**D**) Simpson index, (**E**) PCoA. * represents significant differences vs. K88 group (*: *p* < 0.05, **: *p* < 0.01, ***: *p* < 0.001).

The colonic microbiota of each group was detected by MiSeq high-throughput sequencing to evaluate whether the probiotics could regulate the intestinal microbiota disorders caused by K88. The rarefaction curves of all groups were flattened, which implied that the sequencing depth was sufficient for the analysis of the real situation of the bacterial community (Figure 5A). The alpha diversity was measured by the Chao, Shannon and Simpson indexes. Compared with the CON group, the Chao and Shannon indexes significantly declined in the K88 group (*p* < 0.01), but the Simpson index was the opposite (Figure 5B–D). Compared with the K88 group, the Chao and Shannon indexes in the BK, LK, PK and MK groups were significantly increased (*p* < 0.05), yet the Simpson index in the BK and MK groups were decreased (*p* < 0.05). Furthermore, we used the principal coordinates analysis (PCoA) to analyze the beta diversity of each group, where it was revealed that the K88 group was obviously distinct from the CON group, while the probiotics groups were similar to the CON group (Figure 5E).

**Figure 6:**
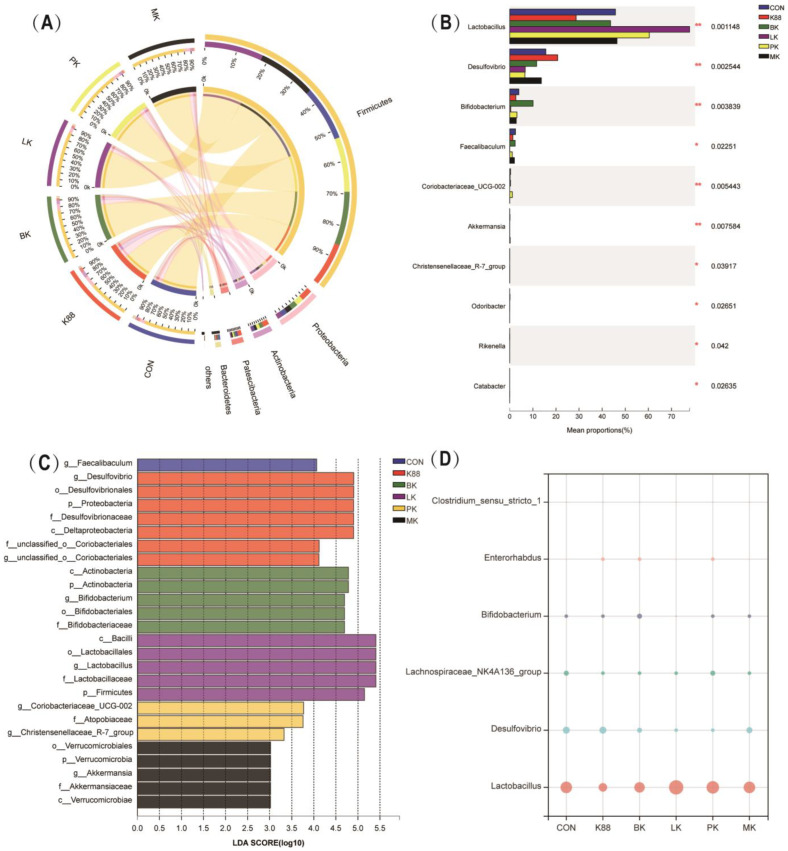
Effect of probiotics on intestinal microbiota composition. (**A**) Microbial distribution at the phylum level, (**B**) species difference analysis at genus level, (**C**) distribution histogram based on LDA, with a log LDA score above 3.0. Significant taxa are labeled and annotated with tags in the right panel. (**D**) Relative abundance of *Lactobacillus*, *Desulfovibrio*, *Lachnospiraceae_NK4A136_group*, *Bifidobacterium*, *Enterorhabdus* and *Clostridium_sensu_stricto_1*. * represents significant differences between the groups (*: *p* < 0.05, **: *p* < 0.01).

At the phylum level, Firmicutes, Proteobacteria, Actinobacteriota, Patescibacteria and Bacteroidetes dominated the intestinal microbiota of CON mice. The K88 group reduced the abundance of Firmicutes and Bacteroidetes, while Proteobacteria, Actinobacteriota and Patescibacteria increased; interestingly, the probiotics alleviated this trend and changed the composition of intestinal microbiota (Figure 6A). Further, we analyzed the top 10 most abundant genera at the genus level, as shown in Figure 6B. Compared with the CON group, the K88 group increased the abundance of *Desulfovibrio* and decreased the abundance of *Lactobacillus*, *Coriobacteriaceae_UCG-002*, *Bifidobacterium*, and *Faecalibaculum* in colon microbiota. However, some of these probiotic groups reversed this phenomenon. Compared with the K88 group, the BK, LK and PK groups had a decrease in *Desulfovibrio*, and a significant increase in *Lactobacillus* abundance was observed. Specifically, BK increased the abundance of *Bifidobacterium*. It is also worth noting that the PK group increased the abundance of *Coriobacteriaceae_UCG-002* and *Christensenellaceae_R-7_group*. Most important of all, MK decreased the abundance of *Desulfovibrio* and apparently increased *Lactobacillus* and *Akkermansia* after the K88 infection. Meanwhile, based on the linear discriminant analysis (LDA) effect size (LEfSe), we learned there were significant differences between the taxonomies (Figure 6C). We extended the LDA score (LDA > 3.0) histogram to uncover the dominant microbiota in each group based on the statistical significance. The relative abundance bubble chart of those representative species indicated that the relative abundance of *Desulfovibrio*, *Enterorhabdus* and *Clostridium_sensu_stricto_1* increased in the K88 group compared with the CON group, but the relative abundance of *Lactobacillus*, *Lachnospiraceae_NK4A136_group* and *Bifidobacterium* showed the opposite status. Compared with the ETEC group, mice that were pre-treated with BK and LK showed an increased abundance of *Bifidobacterium* and *Lactobacillus* (*p* < 0.05) in the intestinal microbiota, while the relative abundance of *Desulfovibrio*, *Enterorhabdus* and *Clostridium_sensu_stricto_1* decreased (Figure 5D).

### 3.6. Bacterial Metabolism of Probiotics Pre-Treatment

**Figure 7:**
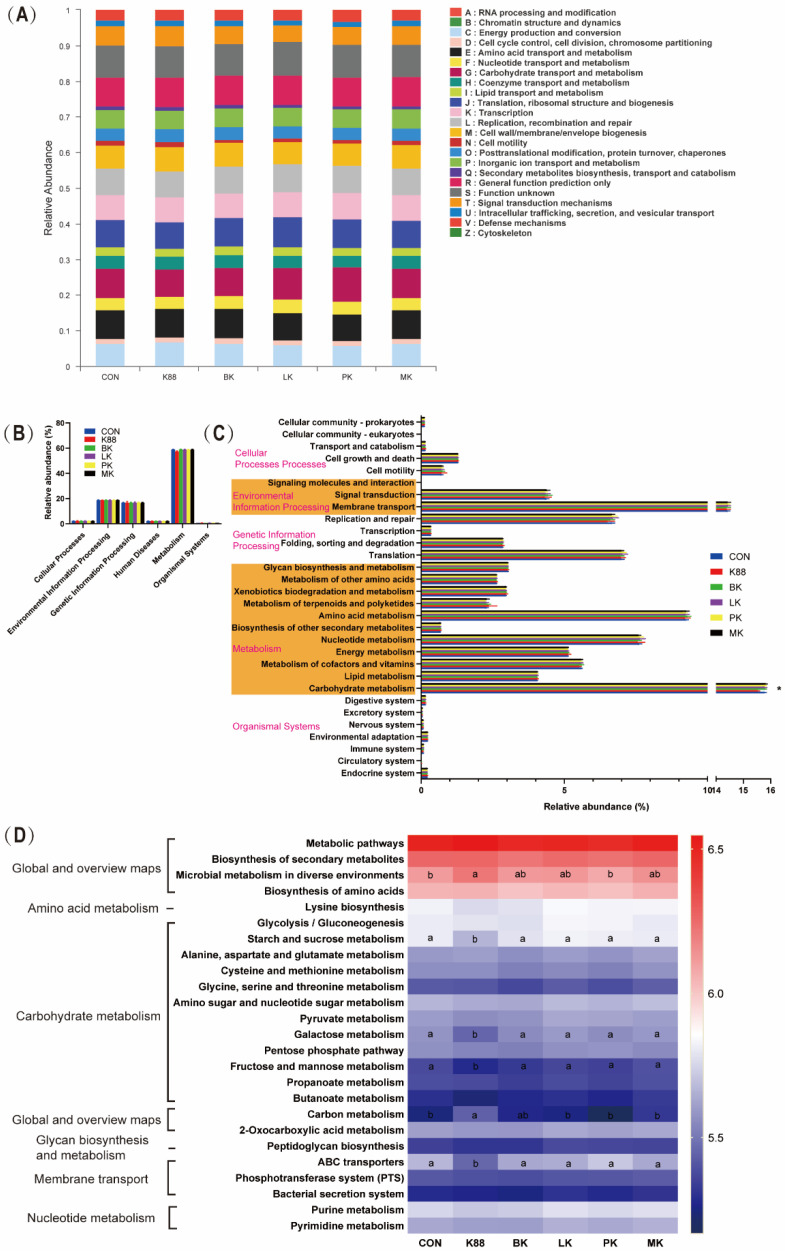
Dynamic bacterial functional profiles analyzed by PICRUSt (*n* = 6). (**A**) COG function classification, (**B**) metabolic pathways in level 1, (**C**) KEGG ortholog functional predictions (Level 2), (**D**) KEGG ortholog functional predictions of the relative abundances of top 25 metabolic functions (Level 3). * Means *p* < 0.05 for CON, BK, LK, PK, MK vs. K88. a, b Means with a row with different superscripts significantly differ (*p* < 0.05).

Figure 7 shows the metabolic functions of microbes according to the Clusters of Orthologous Groups of Proteins (COG) and Kyoto Encyclopedia of Genes and Genomes (KEGG) pathways. Figure 7A shows the changes in COGs in six different groups. Based on the Phylogenetic Investigation of Communities by Reconstruction of Unobserved States (PICRUSt2) function prediction, Figure 7B displayed the microbial gene functions’ plot for bacteria at the first level. As shown in Figure 7B, metabolism accounts for about 60%, genetic information processing about 19% and environmental information processing about 17%. In the K88 group, there was a decrease in metabolism but no significance compared with the other groups in level 1. Going on the above analysis, we found that carbohydrate metabolism had a great improvement compared with the K88 group (*p* < 0.05), which occupied more than 15.6% of the enriched pathways for the probiotics groups (Figure 7C). Furthermore, level 3 of the microbial gene function showed that the microbial metabolism in diverse environments and carbon metabolism were remarkably enriched in the K88 group (*p* < 0.05). Compared with the K88 group, starch and sucrose metabolism, galactose metabolism, fructose metabolism, mannose metabolism and ABC transporters increased with probiotics treatment (*p* < 0.05).

## 4. Discussion

ETEC is one of the most common pathogenic microorganisms associated with food intoxication. Meanwhile, ETEC K88 is among the most important agents causing post-weaning diarrhea in piglets, which causes great damage to the development of the farming industry. It is well known that long-term use of antibiotics can cause numerous side effects; thus, searching for antibiotic alternatives has gradually formed a consensus among the scientific community. Recently, research showed that probiotics exert antimicrobial activity to relieve diarrhea by different mechanisms to control gastrointestinal infections, such as releasing organic acids and bacteriocins [32]. Bifidobacterium and Lactobacillus are both widely distributed in dairy products and act as probiotics in both human and animal gastrointestinal tracts. Our group showed that a microecological preparation could significantly reduce the rate of piglet diarrhea. Therefore, we constructed the ETEC K88-infected mouse model to explore whether probiotics can be used against diarrhea made by ETEC and separately gavaged the probiotics to compare the effect of infection prevention in mice. Subsequently, we measured its function by analyzing the structure of the intestinal mucosa, the expression of inflammation-related genes, the inflammation response and the microbiome of the intestine.

In our study, the mice were curling, shivering and losing weight, while they did not suffer from diarrhea in the K88 group, which is similar to the previous reports [33]. The reasons for this phenomenon include the differences in handling methods and virulence factors of the strains, the sensitivity of the animal models, the number of infections and their duration [34]. Thus, our research showed that lesions within the body manifest as an increased spleen index, decreased colon length, and jejunal pathology. The villus height/crypt depth reflects the absorption capacity of the nutrients in the small intestine. A high ratio represents the structural integrity of the small intestinal villi and their ability to absorb nutrients, while a low ratio represents damage to the intestinal villi and poor absorption ability [35]. In the ETEC-infected group, mice showed epithelial ulceration, and the villus height/crypt depth of the jejunum was markedly reduced, which was consistent with the results of X. Han et al. [36]. However, the probiotic treatment reversed these results, especially in the PK and MK groups. Consistently, it is reported that both Lactobacillus plantarum GL17 and Lactobacillus brevis AY858 can increase the height of villi, reduce the depth of the recess, and alleviate the intestinal injury in mice caused by ETEC [37].

Inflammatory responses are intended to disarm or destroy invaded pathogeny, remove irritants and prepare for tissue repair or wound healing in a short time [38]. Virulence factors of ETEC can, however, cause tissue damage or injury if the immune response is excessive [39]. For example, an ETEC K88 infection significantly increased the concentration of pro-inflammatory cytokines, such as IL-6, IFN-γ and TNF-α in mice serum [40]. A replication of recently identified probiotics supplementation reduced inflammation, where a microencapsulated probiotic combination of *Lactiplantibacillus plantarum* and *Pediococcus acidilactici* was found to reduce pro-inflammatory cytokines (IL-1α, IL-6, IL-8, and TNF-α), which may lead to immune disorders, inflammation and the destruction of the membrane barrier [41]. Our study, as expected, had the same results. Concurrently, we measured the content of immunoglobulin in mice serum. Immunoglobulins (Ig) are important products of immune responses working against antigen materials for organisms, reflecting the immune status of the system [42]. Studies have shown that the *Lactobacillus reuteri* HCM2 group has higher serum IgG and IgA levels than the ETEC group in mice [43], similar to our results. The three probiotics showed strain independence in regulating the levels of inflammatory cytokines and immunoglobulins in different degrees to alleviate the inflammatory reaction caused by ETEC.

We further explored the mechanism by which probiotics regulate intestinal health by examining the expression profiles of the critical genes associated with inflammation in the jejunum of mice. ETEC K88 tends to colonize the jejunum and ileum and causes intestinal damage [44]. In our research, probiotics mitigate this trend, as shown in serum, which conformed to *Pediococcus pentosaceus* L1 in IPEC-J2 IEC [45]. The TLR4, MyD88 and NF-κB are the classic signaling proteins involved in the inflammatory signal transduction system of inflammatory responses [46]. Their expression levels are dramatically elevated, resulting in an increase in the production of inflammatory cytokines such as IL-1β and TNF-α on bacterial infections in mammals [47]. H. Li showed that the *Clostridium butyricum* pre-treatment attenuated the ETEC K88-induced activation of TLR4, MyD88 and NF-κB in weaned piglets [48]. Another report, *Lactobacillus plantarum* BSGP201683, significantly decreased the expression of *TLR4* and *MyD88* in the jejunum of mice [33]. Our results were matched to these classical conclusions.

The intestinal microbiota is a type of dynamic and balanced microecosystem that plays a key role in regulating health [49]. Under normal circumstances, the intestinal microbiota is in balance; however, ETEC K88 secreted endotoxin after colonization in the small intestine [50], resulting in an imbalance of intestinal microbiota and diarrhea. Our results displayed that an ETEC K88 infection led to a significant decrease in colon microbial diversity and richness. The presence of *Lactobacillus plantarum* prevented ETEC from ruining intestinal microbiota diversity [51]. The PCoA analysis revealed that the intestinal microbiota of ETEC K88-infected mice was dramatically different from that of the CON group. Probiotic supplementation can prevent this condition and reverse it to normal. This is not a unique discovery; the function was similar to how *Lactobacillus rhamnosus* ATCC 53103 alleviates the change in bacterial beta diversity caused by EHEC [52]. Combined with the microbial distribution at the phylum level, our findings demonstrated a decrease in Firmicutes and Bacteroidetes and an increase in Proteobacteria and Actinomycetes in the ETEC K88-infected mice, which was in line with a previous study [53]. Firmicutes and Bacteroidetes are the core mice microbiome [54], which have a strong correlation with the host’s health. As the most unstable over time among the four main phyla in the intestinal microbiota, there are many opportunistic pathogens in Proteobacteria, including *Escherichia coli*, *Salmonella*, *Campylobacter* and *Helicobacter*, and their increase can be viewed as an indicator of intestinal diseases [55,56]. At the genus level, *Lactobacillus* and *Bifidobacterium* declined, while *Desulfovibrio* increased in the ETEC K88 group. The *Desulfovibrio* genus is the most often detected in individuals with inflammatory bowel disease (IBD) [57]. Interestingly, not only did the genus cause apoptosis in colon epithelial cells, but it also had a synergistic effect with *E. coli* [58]. These pieces of evidence show that a significant increase in the *Desulfovibrio* genus in the K88 group is closely related to the existence of ETEC K88. *Bifidobacterium* and *Lactobacillus* were found high in the BK and LK groups, and this phenomenon may indicate that the two *Autochthonous Microbiota* have successfully colonized the intestinal tract of four-week-old mice. In the PK group, we found *Coriobacteriaceae_UCG-002* and *Christensenellaceae_R-7_group* significantly different from other groups. Recent studies have demonstrated that *Coriobacteriaceae UCG 002* exerts anti-inflammatory properties and maintains intestinal homeostasis. It produced high concentrations of short-chain fatty acids (SCFAs), which activated the expression of P-glycoprotein [59,60]. *Christensenellaceae_R-7_group*, as a part of the *Christensenellaceae*, demonstrates that its abundance is inversely related to hosting IBD [61,62]. The dominant species (LDA > 3.0) of the MK group was *Akkermansia*, which degrades the excess mucus produced by the inner walls of the intestine [63]. Moreover, *Akkermansia* improves the intestinal barrier’s function, helping to boost the immune system of the host, and its levels inversely correlate to IBD [64]. The abundance of *Akkermansia* has been found to decrease significantly in mice and patients with IBD, according to a previous study [65]. Our outcome showed that mixed bacteria groups could regulate the abundance of *Akkermansia*, which inhibited the intestinal inflammation induced by ETEC K88. Hence, a biomarker may be developed to detect intestinal inflammation based on the relative abundance of *Akkermansia*.

According to the results of the KEGG gene function analyses (levels 1 to 3) of the bacterial functional profiles analyzed, intestinal microbiota altered the host’s metabolism in health and disease. We found that the K88 group was deficient in carbohydrate metabolism at level 2 compared to the CON group. Further analysis showed the probiotics pre-treatment group had huge treasures in metabolisms, such as starch and sucrose metabolism, galactose metabolism, fructose metabolism, mannose metabolism and ABC transporters. In recent studies, *Bifidobacterium longum subsp. infantis* supplementation significantly modulates the colonic pH by consuming HMOs with producing acetic and lactic acids [66] and is metabolized to produce indole-3-lactic acid to decrease inflammation in the intestinal epithelial cells [67]. We guessed that the colonization of probiotics in the probiotic pre-treatment group could absorb sugars through the ABC transport system [68] and convert them into SCFAs, such as acetic acid and their derivatives, which inhibit enterotoxin from the intestine to blood and protect from enteropathogenic infection [17]. 

Our original aim was to find the essential bacteria that play a key role in microecological preparation in the prevention of ETEC K88-induced diarrhea; however, the three probiotics from the microecological preparations had different effects on preventing the intestinal inflammation caused by ETEC K88, and the MK group was slightly better than the other groups. As is known to all, intestinal microbiota will not only interact with the host but also with each other. These microbe–microbe interactions are complex, such as competition, mutualism, commensalism, amensalism and parasitism [69]. For instance, a mixed bacteria treatment may increase *Akkermansia* via the mutual interactions that generate growth factors or available polysaccharides, which facilitate *Akkermansia* proliferation. Subsequently, *Akkermansia* may produce acetate and propionate, which could improve the immune barrier of the host and inhibit the pathogenic substance.

## 5. Conclusions

In summary, the three probiotics from the microecological preparation showed a strain-dependent protective effect on alleviating ETEC K88-infected intestinal inflammation in a mouse model. First, this study revealed a preventive effect of the three probiotics pre-supplementation on decreasing the spleen index, increasing the colon length, repairing the jejunal morphology, relieving weight loss and restoring the ratio of the jejunum villus height to crypt depth. Second, the probiotics alleviated intestinal inflammation in many different ways. We found that probiotics played an essential role in alleviating an ETEC K88 infection by downregulating the pro-inflammatory cytokines (IL-1β, IL-6 and TNF-α), increasing the level of immunoglobulins (IgA, IgM and IgG), and reducing toll-like receptor mRNA expression (*TLR4*, *MyD88* and *NF-κB*). Third, the probiotics modulated the intestinal microbiota composition wrecked by ETEC K88; *Bifidobacterium longum* pre-supplementation promoted the enrichment of the *Bifidobacterium* genus, *Lactobacillus plantarum* promoted the *Lactobacillus* genus, *Pediococcus acidilactici* promoted the *Coriobacteriaceae_UCG-002* and *Christensenellaceae_R-7_group* genus, and three mixed strains promoted the *Akkermansia* genus. Fourth, the probiotics pre-treatment group increased the metabolic function of starch and sucrose metabolism, galactose metabolism, fructose metabolism, mannose metabolism and ABC transporters.

## Figures and Tables

**Table 1:** Primer sequences for q-PCR.

Gene	Forward Primer Sequence (5′ to 3′)	Reverse Primer Sequence (5′ to 3′)	Annealing Temperature (°C)
β-actin	CTAGGCGGACTGTTACTGAGC	CGCCTTCACCGTTCCAGTTT	60
IL-1β	GCCACCTTTTGACAGTGATGAG	GACAGCCCAGGTCAAAGGTT	60
IL-6	GACAAAGCCAGAGTCCTTCAGA	TGTGACTCCAGCTTATCTCTTGG	60
TNF-α	ATGGCCTCCCTCTCATCAGT	TTTGCTACGACGTGGGCTAC	60
TLR4	TTCAGAACTTCAGTGGCTGGATT	CCATGCCTTGTCTTCAATTGTTT	60
MyD88	GCATGGTGGTGGTTGTTTCTG	GAATCAGTCGCTTCTGTTGG	60
NF-κB	CTGAGCGCCCCTCGCATTTA	CCAGCCCATCTTTCTCAGCA	60

## References

[1] Lozano R., Naghavi M., Foreman K., Lim S., Shibuya K., Aboyans V. (2013). Global and regional mortality from 235 causes of death for 20 age groups in 1990 and 2010: A systematic analysis for the Global Burden of Disease Study 2010. Lancet.

[2] Jiang Z., DuPont H.L. (2017). Etiology of travellers’ diarrhea. J. Travel Med..

[3] GBD 2016 Diarrhoeal Disease Collaborators (2018). Estimates of the global, regional, and national morbidity, mortality, and aetiologies of diarrhoea in 195 countries: A systematic analysis for the Global Burden of Disease Study 2016. Lancet Infect Dis..

[4] Gresse R., Chaucheyras-Durand F., Fleury M.A., Wiele T.V.D., Forano E., Blanquet-Diot S. (2017). Intestinal Microbiota Dysbiosis in Postweaning Piglets: Understanding the Keys to Health. Trends Microbiol..

[5] Zhu Y., Luo Q., Davis S.M., Westra C., Vickers T.J., Fleckenstein J.M. (2018). Molecular Determinants of *Enterotoxigenic Escherichia coli* Heat-Stable Toxin Secretion and Delivery. Infect. Immun..

[6] Alison L.S., Timo V.S., Tom J.B., John W.M. (2008). Effect of weaning age on nursery pig and sow reproductive performance. J. Swine Health Prod..

[7] Sterndale S.O., Evans D.J., Mansfield J.P., Clarke J., Sahibzada S., Abraham S., Miller D.W., Kim J.C., Pluske J.R. (2019). Effect of mucin 4 allele on susceptibility to experimental infection with enterotoxigenic F4 Escherichia coli in pigs fed experimental diets. J. Anim. Sci. Biotechnol..

[8] Zhou H., Zhu J., Zhu G. (2012). Fimbriae of animal-originated enterotoxigenic Escherichia coli—A review. Acta Microbiol. Sin..

[9] Xia P., Song Y., Zou Y., Yang Y., Zhu G. (2015). F4+ enterotoxigenic Escherichia coli (ETEC) adhesion mediated by the major fimbrial subunit FaeG. J. Basic Microbiol..

[10] Broeck W.V.D., Cox E., Oudega B., Goddeeris B.M. (2000). The F4 fimbrial antigen of *Escherichia coli* and its receptors. Vet. Microbiol..

[11] Goldenberg J.Z., Yap C., Lytvyn L., Lo C.K., Beardsley J., Mertz D., Johnston B.C. (2017). Probiotics for the prevention of Clostridium difficile-associated diarrhea in adults and children. Cochrane Database Syst. Rev..

[12] Maltz C., Miskovitz P.F., Hajifathalian K. (2020). Lactulose may reduce Clostridium difficile-related diarrhea among patients receiving antibiotics. JGH Open.

[13] Huang G., Li X., Lu D., Liu S., Suo X., Li Q., Li N. (2018). Lysozyme improves intestinal performance and protects against enterotoxigenic Escherichia coli infection in neonatal piglets. Vet. Res..

[14] Ahmed S.T., Hwang J.A., Hoon J., Mun H.S., Yang C.J. (2014). Comparison of single and blend acidifiers as alternative to antibiotics on growth performance, fecal microflora, and humoral immunity in weaned piglets. Asian Austral. J. Anim..

[15] Abiala M., Olayiwola J., Babatunde O., Aiyelaagbe O., Akinyemi S. (2016). Evaluation of therapeutic potentials of plant extracts against poultry bacteria threatening public health. BMC Complem. Altern. Med..

[16] Wang J., Dou X., Song J., Lyu Y., Zhu X., Xu L., Li W., Shan A.-S. (2019). Antimicrobial peptides: Promising alternatives in the post feeding antibiotic era. Med. Res. Rev..

[17] Fukuda S., Toh H., Hase K., Oshima K., Nakanishi Y., Yoshimura K., Tobe T., Clarke J.M., Topping D.L., Suzuki T. (2011). Bifidobacteria can protect from enteropathogenic infection through production of acetate. Nature.

[18] Hrala M., Bosák J., Micenková L., Křenová J., Lexa M., Pirková V., Tomáštíková Z., Koláčková I., Šmajs D. (2021). *Escherichia coli* Strains Producing Selected Bacteriocins Inhibit Porcine *Enterotoxigenic Escherichia coli* (ETEC) under both In Vitro and In Vivo Conditions. Appl. Environ. Microb..

[19] Bermudez-Brito M., Plaza-Díaz J., Muñoz-Quezada S., Gómez-Llorente C., Gil A. (2012). Probiotic mechanisms of action. Ann. Nutr. Metab..

[20] van Zyl W.F., Deane S.M., Dicks L. (2020). Molecular insights into probiotic mechanisms of action employed against intestinal pathogenic bacteria. Intest. Microbes.

[21] Ni X. (2021). Application Progress of Autochthonous Microbiota in Broiler Feed. Feed Indus..

[22] Duranti S., Lugli G.A., Milani C., James K., Mancabelli L., Turroni F., Alessandri G., Mangifesta M., Mancino W., Ossiprandi M.C. (2019). *Bifidobacterium bifidum* and the infant intestinal microbiota: An intriguing case of microbe-host co-evolution. Environ. Microbiol..

[23] O’Callaghan A., van Sinderen D. (2016). Bifidobacteria and their role as members of the human intestinal microbiota. Front. Microbiol..

[24] Wu T., Shi Y., Zhang Y., Zhang M., Zhang L., Ma Z., Zhao D., Wang L., Yu H., Hou Y. (2021). *Lactobacillus rhamnosus* LB1 Alleviates *Enterotoxigenic Escherichia coli*-Induced Adverse Effects in Piglets by Improving Host Immune Response and Anti-Oxidation Stress and Restoring Intestinal Integrity. Front. Cell. Infect. Microbiol..

[25] Yang B., Yue Y., Chen Y., Ding M., Li B., Wang L., Wang Q., Stanton C., Ross R.P., Zhao J. (2021). *Lactobacillus plantarum* CCFM1143 Alleviates Chronic Diarrhea via Inflammation Regulation and Intestinal Microbiota Modulation: A Double-Blind, Randomized, Placebo-Controlled Study. Front. Immunol..

[26] Tan K., Deng D., Ma X., Cui Y., Tian Z. (2020). Pediococcus acidilactici P25 Protected Caenorhabditis elegans against *Enterotoxigenic Escherichia coli* K88 Infection and Transcriptomic Analysis of Its Potential Mechanisms. Biomed. Res. Int..

[27] Mao X., Gu C., Hu H., Tang J., Chen D., Yu B., He J., Yu J., Luo J., Tian G. (2016). Dietary Lactobacillus rhamnosus GG Supplementation Improves the Mucosal Barrier Function in the Intestine of Weaned Piglets Challenged by Porcine Rotavirus. PLoS ONE.

[28] Xue C., Li Y., Lv H., Zhang L., Bi C., Dong N., Shan A., Wang J. (2021). Oleanolic Acid Targets the Intestinal-Liver Axis to Alleviate Metabolic Disorders and Hepatic Steatosis. J. Agric. Food Chem..

[29] Xue C., Lv H., Li Y., Dong N., Wang Y., Zhou J., Shi B., Shan A. (2022). Oleanolic acid reshapes the intestinal microbiota and alters immune-related gene expression of intestinal epithelial cells. J. Sci. Food Agric..

[30] Aluri J., Cooper M.A., Schuettpelz L.G. (2021). Toll-Like Receptor Signaling in the Establishment and Function of the Immune System. Cells.

[31] Damien P., Cognasse F., Payrastre B., Spinelli S.L., Blumberg N., Arthaud C.A., Eyraud M.A., Phipps R.P., McNicol A., Pozzetto B. (2017). NF-κB Links TLR2 and PAR1 to Soluble Immunomodulator Factor Secretion in Human Platelets. Front. Immunol..

[32] Peters V.B.M., van de Steeg E., van Bilsen J., Meijerink M. (2019). Mechanisms and immunomodulatory properties of pre- and probiotics. Benef. Microbes.

[33] Liu Q., Ni X.-Q., Wang Q., Peng Z., Niu L., Wang H., Zhou Y., Sun H., Pan K., Jing B. (2017). Lactobacillus plantarum BSGP201683 Isolated from Giant Panda Feces Attenuated Inflammation and Improved Intestinal Microflora in Mice Challenged with *Enterotoxigenic Escherichia coli*. Front. Microbiol..

[34] Bertin A. (1983). Virulence factors of *enterotoxigenic E. coli* studied in the infant mouse model. Ann. Rech. Vet..

[35] Yu Q., Liu X., Liu Y., Riederer B., Li T., Tian D., Tuo B., Shull G., Seidler U. (2016). Defective small intestinal anion secretion, dipeptide absorption, and intestinal failure in suckling NBCe1-deficient mice. Pflugers Arch..

[36] Lin Q., Fu Q., Li X., Luo Y., Luo J., Chen D., Mao X., Yu B., Zheng P., Huang Z. (2021). Human β-Defensin 118 Attenuates *Escherichia coli* K88-Induced Inflammation and Intestinal Injury in Mice. Probiotics Antimicrob. Proteins.

[37] Han X., Ding S., Ma Y., Fang J., Jiang H., Li Y., Liu G. (2021). Lactobacillus plantarum and Lactobacillus brevis Alleviate Intestinal Inflammation and Microbial Disorder Induced by ETEC in a Murine Model. Oxid. Med. Cell. Longev..

[38] Ren W., Yin J., Duan J., Liu G., Zhu X., Chen S., Li T., Wang S., Tang Y., Hardwidge P.R. (2014). Mouse intestinal innate immune responses altered by *Enterotoxigenic Escherichia coli* (ETEC) infection. Microbes Infect..

[39] Sanchez-Villamil J., Navarro-Garcia F. (2015). Role of virulence factors on host inflammatory response induced by diarrheagenic *Escherichia coli* pathotypes. Future Microbiol..

[40] Liu B., Liu Q., Li G., Sun L., Gao Y., Zhang Y., Liu H., Cao M., Liu G. (2019). The anti-diarrhea activity of red algae-originated sulphated polysaccharides on ETEC-K88 infected mice. RSC Adv..

[41] Pupa P., Apiwatsiri P., Sirichokchatchawan W., Pirarat N., Nedumpun T., Hampson D., Muangsin N., Prapasarakul N. (2022). Microencapsulated probiotic Lactiplantibacillus plantarum and/or Pediococcus acidilactici strains ameliorate diarrhoea in piglets challenged with *Enterotoxigenic Escherichia coli*. Sci. Rep..

[42] Megha K., Mohanan P. (2021). Role of immunoglobulin and antibodies in disease management. Int. J. Bio. Macromol..

[43] Wang T., Teng K., Liu G., Liu Y., Zhang J., Zhang X., Zhang M., Tao Y., Zhong J. (2018). Lactobacillus reuteri HCM2 protects mice against *Enterotoxigenic Escherichia coli* through modulation of intestinal microbiota. Sci. Rep..

[44] Fairbrother J., Nadeau E., Gyles C. (2005). *Escherichia coli* in postweaning diarrhea in pigs: An update on bacterial types, pathogenesis, and prevention strategies. Anim. Health Res. Rev..

[45] Yin H., Ye P., Lei Q., Cheng Y., Yu H., Du J., Pan H., Cao Z. (2020). In Vitro probiotic properties of Pediococcus pentosaceus L1 and its effects on enterotoxigenic Escherichia coli-induced inflammatory responses in porcine intestinal epithelial cells. Microb. Pathog..

[46] Zeytun A., Chaudhary A., Pardington P., Cary R., Gupta G. (2010). Induction of cytokines and chemokines by Toll-like receptor signaling: Strategies for control of inflammation. Crit. Rev. Immunol..

[47] Zughaier S., Zimmer S., Datta A., Carlson R., Stephens D. (2005). Differential induction of the toll-like receptor 4-MyD88-dependent and -independent signaling pathways by endotoxins. Infect Immun..

[48] Li H., Liu X., Shang Z., Qiao J. (2021). *Clostridium butyricum* helps to alleviate inflammation in weaned piglets challenged with *Enterotoxigenic Escherichia coli* K88. Front. Vet. Sci..

[49] Yang X., Xiao Z., Liu F., Chen S., Tang W., Zhang D., Liu S. (2016). *Enterotoxigenic Escherichia coli* infection alters intestinal immunity in mice. Mol. Med. Rep..

[50] Kataoka K. (2016). The intestinal microbiota and its role in human health and disease. J. Med. Investig..

[51] Yue Y., He Z., Zhou Y., Ross R.P., Stanton C., Zhao J., Zhang H., Yang B., Chen W. (2020). *Lactobacillus plantarum* relieves diarrhea caused by enterotoxin-producing *Escherichia coli* through inflammation modulation and intestinal microbiota regulation. Food Funct..

[52] Hu Y., Zhao M., Lu Z., Lv F., Zhao H., Bie X., Johnsonii L. (2021). *L. plantarum*, and *L. rhamnosus* alleviated Enterohaemorrhagic *Escherichia coli*-induced diarrhoea in mice by regulating intestinal microbiota. Microb. Pathog..

[53] Li Y., Liao Q., Lin M., Zhong D., Wei L., Han B., Miao H., Yao M., Xie Z. (2015). An integrated metabonomics and microbiology analysis of host-microbiota metabolic interactions in rats with coptis chinensis-induced diarrhea. RSC Adv..

[54] Sun X., Gao Y., Wang X., Hu G., Wang Y., Feng B., Hu Y., Mu X., Zhang Y., Dong H. (2019). *Escherichia coli* O101-induced diarrhea develops intestinal microbial dysbiosis in rats. Exp. Ther. Med..

[55] Rizzatti G., Lopetuso L.R., Gibiino G., Binda C., Gasbarrini A. (2017). Proteobacteria: A Common Factor in Human Diseases. Biomed. Res. Int..

[56] Shin N.R., Whon T.W., Bae J.W. (2015). Proteobacteria: Microbial signature of dysbiosis in intestinal microbiota. Trends Biotechnol..

[57] Kushkevych I., Dordević D., Kollár P. (2019). Analysis of Physiological Parameters of Desulfovibrio Strains from Individuals with Colitis. Open Life Sci..

[58] Coutinho C., Coutinho-Silva R., Zinkevich V., Pearce C.B., Ojcius D.M., Beech I. (2017). Sulphate-reducing bacteria from ulcerative colitis patients induce apoptosis of gastrointestinal epithelial cells. Microb. Pathog..

[59] Guo W., Mao B., Cui S., Tang X., Zhang Q., Zhao J., Zhang H. (2022). Protective effects of a novel probiotic *Bifidobacterium pseudolongum* on the intestinal barrier of colitis mice via modulating the Pparγ/STAT3 pathway and intestinal microbiota. Foods.

[60] Van der Beek C.M., Dejong C.H.C., Troost F.J., Masclee A.A.M., Lenaerts K. (2017). Role of short-chain fatty acids in colonic inflammation, carcinogenesis, and mucosal protection and healing. Nutr. Rev..

[61] Waters J.L., Ley R.E. (2019). The human intestinal bacteria Christensenellaceae are widespread, heritable, and associated with health. BMC Biol..

[62] Mancabelli L., Milani C., Lugli G.A., Turroni F., Cocconi D., van Sinderen D., Ventura M. (2017). Identification of universal intestinal microbial biomarkers of common human intestinal diseases by meta-analysis. FEMS Microbiol. Ecol..

[63] Depommier C., Everard A., Druart C., Plovier H., Van Hul M., Vieira-Silva S., Falony G., Raes J., Maiter D., Delzenne N.M. (2019). Supplementation with Akkermansia muciniphila in overweight and obese human volunteers: A proof-of-concept exploratory study. Nat. Med..

[64] Macchione I.G., Lopetuso L.R., Ianiro G., Napoli M., Gibiino G., Rizzatti G., Petito V., Gasbarrini A., Scaldaferri F. (2019). *Akkermansia muciniphila*: Key player in metabolic and gastrointestinal disorders. Eur. Rev. Med. Pharmacol. Sci..

[65] Wang L., Tang L., Feng Y., Zhao S., Han M., Zhang C., Yuan G., Zhu J., Cao S., Wu Q. (2020). A purified membrane protein from *Akkermansia muciniphila* or the pasteurised bacterium blunts colitis associated tumourigenesis by modulation of CD8(+) T cells in mice. Intestinal.

[66] Frese S.A., Hutton A.A., Contreras L.N., Shaw C.A., Palumbo M.C., Casaburi G., Xu G., Davis J.C.C., Lebrilla C.B., Henrick B.M. (2017). Persistence of Supplemented *Bifidobacterium longum subsp. infantis* EVC001 in Breastfed Infants. mSphere.

[67] Ehrlich A.M., Pacheco A.R., Henrick B.M., Taft D., Xu G., Huda M.N., Mishchuk D., Goodson M.L., Slupsky C., Barile D. (2020). Indole-3-lactic acid associated with Bifidobacterium-dominated microbiota significantly decreases inflammation in intestinal epithelial cells. BMC Microbiol..

[68] Jeckelmann J.M., Erni B. (2020). Transporters of glucose and other carbohydrates in bacteria. Pflugers Arch..

[69] Smid E.J., Lacroix C. (2013). Microbe-microbe interactions in mixed culture food fermentations. Curr. Opin. Biotechnol..

